# The Potential of Non-ablative Erbium (YAG) Laser Treatment for Complications After Midurethral Sling Surgery: A Narrative Review

**DOI:** 10.7759/cureus.58486

**Published:** 2024-04-17

**Authors:** Nobuo Okui

**Affiliations:** 1 Dentistry, Kanagawa Dental University, Kanagawa, JPN; 2 Urology, Yokosuka Urogynecology and Urology Clinic, Kanagawa, JPN

**Keywords:** mesh exposure, vel, non-ablative erbium (yag) laser, stress urinary incontinence, midurethral sling

## Abstract

Midurethral sling (MUS) surgery, using tension-free vaginal tape and transobturator tape, has been widely adopted for the treatment of stress urinary incontinence (SUI). However, postoperative complications, including persistent urinary incontinence, mesh exposure, and pain, have become problematic, and surgical treatments for these complications face challenges, such as invasiveness, treatment-resistant cases, and recurrence. This review provides an overview of the current evidence regarding these complications and the potential of vaginal non-ablative erbium (YAG) laser (VEL) treatment as a minimally invasive option with low risk of complications. Studies have suggested the effectiveness of VEL treatment, performed using devices such as IncontiLase (SP Dynamis; Fotona d.o.o., Ljubljana, Slovenia), for persistent urinary incontinence after MUS surgery, pain following mesh removal, and asymptomatic mesh exposure. VEL treatment is expected to be a new treatment option for complications following MUS surgery; however, further large-scale comparative trials are required to verify its efficacy and safety and to establish criteria for its indications. Appropriate assessment of the indications and provision of sufficient information to patients is important when presenting VEL as a treatment option.

## Introduction and background

Urethral sling surgery, particularly the introduction of midurethral slings (MUS) using polypropylene mesh tapes, has been recognized as a treatment that significantly improves the quality of life (QoL) of patients with stress urinary incontinence (SUI) [1,2]. MUS, performed in the form of tension-free vaginal tape (TVT) or transobturator tape (TOT), offers unique advantages in the management of SUI [3,4]. However, several issues have arisen regarding this treatment, including mesh-related complications, the need for reoperation, international litigation, and national-level investigations [3]. These issues may cause additional distress to patients, particularly concerning the postoperative persistence of SUI, mesh pain, erosion, and exposure [5,6].

Recent advancements in non-ablative erbium (YAG) laser (Er:YAG laser) technology are expected to provide new treatment options for complications after MUS [7]. This laser has a wavelength of 2,940 nm and has a high affinity for water. The Er:YAG laser converts light energy into heat energy that is rapidly absorbed by the upper 5 μm of the epidermis. The treatment mechanism involves two approaches: (1) utilizing an optimal number of pulses and pulse durations to maximize superficial tissue response while achieving moderate coagulation depth and (2) using multiple pulses irradiated in long pulse sequences to achieve a consistent temperature range deep beneath the surface. This results in "smooth resurfacing" and induces biomodulatory effects through thermal shock [8].

The molecular biological mechanism of Er:YAG laser technology was explained by the variable heat shock response model proposed by Lukač et al. [9]. This model focuses on the synergistic action of biochemical processes that regulate cell survival and tissue damage in short- and long-term exposure [10]. Several studies have shown that laser treatment potentially offers a new solution to issues such as mesh-related complications observed after MUS surgery.

This narrative review focuses on complications following urethral sling surgery and their contemporary treatment options, particularly the potential of vaginal non-ablative Er:YAG laser (VEL) treatment. VEL treatment, performed using devices such as IncontiLase (SP Dynamis; Fotona d.o.o., Ljubljana, Slovenia), may contribute to the development of new treatment strategies for SUI management and represent an important step towards further improving patients' QoL.

## Review

Development of MUS surgery

The International Continence Society defines SUI as the complaint of involuntary urine loss due to effort or physical exertion, such as during sporting activities, or resulting from sneezing or coughing [11]. Lee et al. analyzed the Optum® de-identified Clinformatics® Data Mart (CDM) for women aged 18-64 years and the Centers for Medicare and Medicaid Services (CMS) Medicare 5% Sample for women aged 65 years or older to examine trends in the surgical management of female urinary incontinence from 2004 to 2013. During this period, the percentage of women with urinary incontinence who underwent surgical treatment declined from 4.7% to 2.7% in the CMS and from 12.5% to 9.1% in the CDM. MUSs were the most common procedure but started to decline in 2011, ultimately decreasing by approximately 50% during the study period. The prevalence of urinary incontinence-related surgical procedures, including slings, was the highest among women aged 35-54 years and Caucasian women and the lowest among women residing in the northeast region of the United States [12]. Two primary sling varieties exist: the TVT and TOT slings. The TVT variant made its debut in 1996, which was followed by the introduction of the TOT model in the early 2000s [13,14]. Both used a polypropylene mesh tape [15]. The TVT technique involves placing the sling from the midurethra to behind the pubic symphysis, whereas TOT involves placing the sling from the midurethra to the obturator foramen [16]. In 2012, a third type of mini-sling was introduced to further reduce surgical invasiveness [17,18].

Mesh issues in MUS

While MUS has been widely used, concerns have been raised regarding the lack of safety data as part of patient information and medical decision-making [19]. Data on the frequency of follow-up surgeries extending beyond a five-year period post-implantation are insufficient [20]. In 2007, a systematic review and meta-analysis of the short-term safety of MUS was conducted, but it did not include an analysis of reoperation rates or complications beyond two years [21]. Complications associated with mesh surgery have been recognized as a problem with the use of polypropylene mesh for pelvic organ prolapse repair, but MUS carries similar risks, triggering international litigation against manufacturers and national-level investigations. Gurol-Urganci et al. [20] reported that the use of midurethral slings in the UK decreased rapidly, with a reduction of approximately 50% between 2008 and 2017. This suggests a growing concern regarding the mesh. In fact, TOT and TVT have been excluded from guidelines in some countries because of insurance coverage issues [19]. Artsen et al. analyzed 34,485 reports in the FDA Adverse Event Reporting Database and identified pain (>90%), exposure (>90%), and infection as the main complications of mesh surgery [22]. Karim et al. conducted a systematic review of 20 studies (198 patients) from 1996 to 2018 and reported mesh erosion after incontinence surgery. The most common primary surgeries were TVT or TOT, accounting for 75% of the total surgeries, and the sites of mesh erosion were the bladder in 68% (134 patients), bladder neck in 6% (12 patients), and urethra in 32% (63 patients) [23]. In a review of 1,439 cases by Kokanali et al., mesh exposure was observed in 4.7% (41 cases) after TOT and 3.5% (20 cases) after TVT, with risk factors including advanced age (P <0.05), diabetes (odds ratio: 3.4), smoking (odds ratio: 4.2), vaginal incision length >2 cm (odds ratio: 4.4), postoperative complications (odds ratio: 6.9), and a history of pelvic organ prolapse or urinary incontinence surgery (odds ratio: 2.3) [24]. Wijffels et al. reported transurethral resection for three cases of urethral mesh exposure and proposed it as a minimally invasive and effective treatment method [25]. Gurol-Urganci et al. reported that, among women who underwent midurethral sling surgery for stress urinary incontinence, the mesh sling removal rate over nine years was 3.3% [20]. This finding suggests that the removal rate may be lower than the incidence of complications. They also reported that 99.3% of women who had a retropubic midurethral sling inserted for the first time between April 1, 2006, and December 31, 2015, still had complete or partial mesh sling in place at the end of the study period [20]. This implies that there may be patients who are unsure of how to proceed when complications arise because of the lack of guidelines for managing such situations.

Differences in mesh issues between TVT and TOT

When comparing TOT and TVT, certain differences in complication profiles were observed. In a comparative trial of 615 cases by Chae et al., there was no significant difference in postoperative complication rates between the TOT and TVT groups (14.4% vs. 15.5%), but more groin and thigh pain were reported with TOT [26]. A meta-analysis of 12 randomized comparative trials by Latthe et al. also found significantly more groin and thigh pain with TOT than with TVT (odds ratio: 8.05, 95%CI: 3.78-17.16) [27]. On the other hand, a review by Long et al. suggested that TVT tends to have slightly more postoperative urinary retention than TOT (odds ratio: 1.6; 95%CI: 0.90-3.12; p=0.06), and based on ultrasound and urodynamic findings, TVT is considered to have higher obstructiveness [28]. However, Latthe et al.'s meta-analysis found no significant difference in postoperative urinary retention rates (TOT odds ratio: 0.61, 95%CI: 0.35-1.07; TVT odds ratio: 0.81, 95%CI: 0.48-1.31), and bladder injury (TOT odds ratio: 0.11, 95%CI: 0.05-0.25) and hematoma (TOT odds ratio: 0.06, 95%CI: 0.01-0.30) were significantly less frequent with TOT [27]. Mesh exposure rates were similar between TOT (odds ratio: 1.11, 95%CI: 0.54-2.28) and TVT (odds ratio: 0.77, 95%CI: 0.22-2.72) [27].

Mechanisms of mesh issues

Several studies have reported on the pathological mechanisms underlying mesh issues. Wang et al. stated that degraded and exfoliated polypropylene mesh due to aging induces a significant inflammatory response in the surrounding tissue [24]. In severely degraded mesh, M2-type macrophages and T lymphocytes increased, showing an increasing trend with the degree of degradation [24]. Taylor et al. focused on the friction-induced erosion of adjacent soft tissues by the mesh material in "mesh erosion," a known postoperative complication, and reported erosion rates consistent with clinical experience [15]. Okui et al.'s study on the classification of mesh fixation status considered both pathological mechanisms and three-dimensional positioning. They classified the complications of polypropylene mesh in LSC into three groups based on the fixation status of the mesh [29]. Group I included cases where the sacral fixation was detached, the mesh adhered in an unintended position, and cell regeneration was inhibited in the gaps of the overlapped mesh. Group II consisted of cases in which mesh tension at the sacral fixation site was excessive, hindering tissue regeneration. The findings in Groups I and II suggest problems with the pathological issues of the mesh and the surgical technique itself. Group III included cases in which the mesh was fixed in the intended position but had aged over time, with cracks in the surrounding tissue due to the aging of the mesh fibers, promoting the development of unhealthy granulation tissue and revealing pathological problems related to the material properties of the mesh [29]. This classification may also be applied to midurethral slings, such as TVT and TOT. In other words, the fixation status and age-related degradation of the mesh in the midurethral slings may lead to persistent SUI, pain, and exposure after surgery. Similarly, in Okui et al.'s tissue study of mesh erosion, fiber degradation progressed with a longer mesh placement duration, and abrasions and cracks were observed in the surrounding area [30]. Additionally, vacuoles along the mesh shape fuse and enlarge, forming abscesses or hematomas. However, if cell proliferation around the vacuoles is confirmed, bleeding and pain may improve [30].

Complications after midurethral sling surgery and surgical intervention

Minimally invasive surgery is an option for complications following TVT or TOT surgery. However, in cases of refractory mesh exposure or severe urethral stricture, surgical mesh removal or urethroplasty may be necessary [31]. In a study by Liapis et al., TVT surgery was performed again in 31 women whose previous TVT or TOT surgery had failed, resulting in persistent or recurrent urinary incontinence. At the two-year postoperative follow-up, 84% of the women showed improvement in urinary incontinence, with objective and subjective cure rates of 77% and 71%, respectively. Complications were few, with bladder perforation in one case and transient voiding difficulty in two cases [32]. On the other hand, Okui et al. reported that some patients who failed TVT surgery felt anxious about having the TVT inserted again [33]. Hou et al. reported on the outcomes of mesh removal in 70 women who underwent mesh removal solely for pain after urethral sling surgery such as TVT/TOT. After removal, pain improved in 94% of women and was completely resolved in 67% of patients. However, 20% of women experience recurrent or worsening urinary incontinence [34]. Rigaud et al. performed mesh excision in 34 women who presented with chronic pelvic pain after TVT or TOT surgery. After mesh excision, pain improved in 71% of women and completely resolved in 50% of women. However, 21% of women had recurrent or worsening urinary incontinence, and 8% developed new urinary urgency [35]. Jeffery et al. investigated outcomes after mesh removal in 63 women who underwent mesh removal for mesh-related complications following transvaginal mesh surgery for pelvic organ prolapse. After mesh removal, mesh erosion recurred in 23% of women, requiring additional surgery. The risk of recurrence was higher when the mesh was partially removed and lower when it was completely removed [36]. Marcus-Braun et al. also analyzed 104 mesh removal surgeries for mesh complications after transvaginal mesh surgery for pelvic organ prolapse. Partial mesh removal was associated with a high recurrence rate of mesh erosion, with 40% of patients requiring reoperation [37]. It has also been reported that sports athletes tend to avoid midurethral slings because of concerns about decreased athletic performance with an artificial object placed in the body [38-40]. Addressing refractory complications requires knowledge of both urology and gynecology, and previous studies suggest that surgical intervention on the mesh is not always successful.

Periurethral collagen injection is an option in cases of persistent SUI after MUS. Corcos et al. followed 65 women for four years who underwent periurethral collagen injection therapy for SUI after previous failed surgery, including TVT. At four years, 60% of patients showed improvement in SUI, and pad tests also showed significant improvement [40]. Bent et al. evaluated the efficacy and safety of periurethral collagen injection in women with SUI and urethral hypermobility. The study included patients who had previously failed SUI surgery. At 12 months, 81% of the patients showed improvement in SUI, and QOL scores also significantly improved [41]. However, the availability of periurethral-specific collagen is limited in certain countries, and not all countries worldwide have access to it [42,43]. In a review by Mahdy et al., the role of urethral bulking agents (such as collagen) in SUI was discussed amid the growing concern over mesh complications. The authors point out that collagen injection therapy lacks long-term efficacy and has a limited supply of injection materials, leading to its restricted use in many countries currently [42]. Herschorn's review mentioned the current status of collagen injection therapy for SUI in Canada. In Canada, collagen injection therapy was introduced in the late 1990s, but its long-term efficacy is limited and the supply of injection materials is unstable [43]. Crivellaro et al.'s review mentions collagen injection therapy as a minimally invasive treatment for SUI [44]. Although collagen injection therapy can provide short-term efficacy, its long-term efficacy is limited. The risk of allergic reactions to collagen and infection at the injection site has also been mentioned [44].

Persistent SUI after TVT/TOT and laser treatment

Recent reports suggest that VEL is effective for urinary incontinence after TVT/TOT. In a study by Erel et al., RenovaLase was used to compare the effects of VEL treatment in 25 cases of urinary incontinence after TVT/TOT and 25 cases of urinary incontinence without TVT/TOT [45]. The initial International Consultation on Incontinence Questionnaire-Urinary Incontinence Short Form (ICIQ-SF) score was significantly higher in the post-TVT/TOT group (16.04 vs 12.76, p=0.013). However, after treatment, both groups showed similarly (5.96 vs 6.04, p=0.921), and the severity of urinary incontinence significantly decreased (p=0.000). Higher treatment efficacy was observed in younger patients (p=0.012) and in those with a shorter post-menopausal period (p=0.011). Furthermore, the good responder group, which showed an improvement of 50% or more, had a significantly longer duration of treatment efficacy than the poor responder group. These results suggest that VEL may be an effective treatment option for urinary incontinence after TVT/TOT [30]. This is also supported by a retrospective study (with a control group) conducted at multiple institutions in Japan using LncontiLase [46]. Japan's retrospective study at multiple institutions compared TVT and VEL and found similar improvements in the one-hour pad test and ICIQ-SF; however, VEL was significantly superior to TVT in OABSS. Analysis after propensity score matching also showed that, although de novo urgency and de novo urge incontinence occurred postoperatively in the TVT group, improvement in overactive bladder (OAB) was observed in the VEL group [31]. Therefore, tissue repair effects of VEL can be expected in cases of persistent urinary incontinence after TVT/TOT.

To capitalize on the strengths of VEL, it is important to properly assess its indications. Erel et al. demonstrated the effectiveness of VEL for urinary incontinence after TVT/TOT, but its efficacy was particularly high in younger patients and those in the early postmenopausal period [45]. In Japan, a retrospective study at multiple institutions showed that VEL was more effective in patients who were sexually active [31]. It has also been shown that VEL was more effective in patients who desire pregnancy [46]. In contrast, Fistonić et al. reported a prospective cohort that explored predictors of short-term efficacy of VEL for SUI. Laser treatment was performed in 85 women, and the results of multivariate logistic regression analysis showed that higher pretreatment International Consultation on Incontinence Questionnaire-Urinary Incontinence (ICIQ-UI) scores, lower BMI, younger age, lower average birth weight, and shorter perineal muscle training duration were independent predictors of improvement in ICIQ-UI scores after laser treatment [47]. Gambacciani et al.'s study examined the long-term effects of VEL and predictors of treatment responsiveness for genitourinary syndrome of menopause. Three laser treatments were performed on 205 women, who were followed up for 12 months. After treatment, improvements in vaginal atrophy symptoms, vaginal pH, and an increase in the vaginal health index were observed, which persisted for 12 months. Age, postmenopausal duration, and BMI were identified as predictors of treatment responsiveness, suggesting that better effects can be obtained in younger women, those in the early postmenopausal period, and those with a lower BMI [48].

In a multicenter blinded randomized sham-controlled trial a total of 110 participants with SUI were randomized to active arm using Er:YAG laser therapy and sham arm using sham handpiece [49]. Patients received two treatments of IncontiLase one month apart. The primary outcomes measure was a one-hour pad weight test measured at six months. Secondary outcomes were durability of treatment success at 12 months and questionnaires for assessment of SUI severity (ICIQ-SF), sexual function (Pelvic Organ Prolapse/Urinary Incontinence Sexual Questionnaire) and Health-Related Quality of Life in King's Health Questionnaire, and incidence and severity of device-related adverse events and pain (visual analog scale: VAS). It was determined that IncontiLase non-ablative vaginal Er:YAG laser therapy significantly improved SUI symptoms versus sham treatment and should be considered as a non-surgical treatment option for SUI.

Mesh excision and laser treatment for mesh pain

VEL is also effective for mesh pain and is expected to have tissue repair effects when combined with mesh removal. Okui et al.'s study on mesh excision and laser treatment underwent histopathological examination to assess the complications of midurethral sling mesh, changes in tissues containing the polypropylene mesh, and the effects of VEL treatment [33]. This study demonstrated the usefulness of mesh removal and laser treatment. Pathological findings before treatment showed vacuole formation and foreign body reactions around the mesh, but one year after mesh removal and VEL treatment, cell regeneration and tissue repair were confirmed. VAS scores showed significant improvements in pain (from 9.0 to 1.22), bleeding (from 8.11 to 0.44), and QOL (from 8.56 to 0.44). On the other hand, Kobashi et al.'s study reported the results of transvaginal mesh removal for polypropylene mesh slings. Of the 20 cases, 15 (75%) had bladder or urethral fistulas, and complete mesh removal resulted in complications such as bleeding and bladder injury in five cases (25%) [50]. It was also mentioned that five cases (25%) had recurrent or worsening urinary incontinence after surgery [50]. Jambusaria et al.'s study reported that, overall, 3.3% of patients required mesh removal or revision, of which complete removal was performed in 53.4%. Postoperative complications included pain (36.8%), urinary incontinence (35.3%), and urgency (19.5%) [51]. Therefore, laser treatment in combination with mesh excision is considered useful for refractory mesh removal [33]. The method used in Okui et al.'s study on mesh excision and laser treatment was a combination of intraurethral (UEL) and VEL treatments using IncontiLase. Erel et al. reported the advantages of UEL + VEL for SUI in postmenopausal women [52]. The study included 122 patients with SUI, and at three months, both groups showed significant improvement in SUI symptoms, with particularly remarkable improvement observed in postmenopausal patients who received UEL+VEL treatment (p=0.003). Gambacciani et al. reported the high safety of VEL performed on over 113,000 patients in 535 institutions across 43 countries over the past eight years [53]. Laser treatment is minimally invasive, does not use a mesh, and has no risk of exposure, making it a promising treatment option. Further research and examination of the indications are required.

Mesh erosion and laser treatment

In a study on mesh erosion and VEL, VEL treatment was administered to nine women (mean age: 73.2 years, mean duration post mesh insertion: 7.2 years) who experienced vaginal mesh erosion, and its effects were evaluated [30]. These patients suffered from vaginal erosion after a polypropylene mesh was used for the treatment of pelvic organ prolapse. Histopathological analysis before treatment confirmed the tissue destruction and inflammatory reactions caused by the mesh. After VEL treatment, the patients experienced significant improvements in bleeding and related symptoms due to erosion, and their QoL improved. Histopathological analysis confirmed cell regeneration and tissue repair, demonstrating the effectiveness of this treatment. The study results suggest that VEL may be an effective method for treating vaginal mesh erosion [30]. In particular, compared with the existing treatment method of physical mesh removal, VEL is considered a safer treatment with expected regenerative effects. However, this study has limitations in generalizing the results due to the small sample size and being based on a retrospective nature of the study.

Importance of long-term follow-up

Long-term follow-up is important after TVT or TOT surgery even in asymptomatic cases. Braga et al. conducted a 20-year follow-up after TVT surgery and found long-term complications, such as mesh exposure (6.3%), de novo urge incontinence (6.9%), and recurrence (12.5%). They emphasized the importance of early detection and treatment intervention through regular symptom assessment and cystoscopy [54]. Khanuengkitkong et al. reported a case of mesh exposure 10 years after TVT surgery and stressed the need for regular follow-up, even in asymptomatic cases [55]. In cases of persistent urinary incontinence or asymptomatic mesh exposure after surgery, VEL may be an effective treatment option.

Psychological burden after mesh surgery

Women who have undergone mesh surgery experience not only physical complications but also psychological burdens, such as limitations in social life and anxiety about the future. Motamedi et al. reported that women's daily lives were greatly changed by mesh surgery, with ongoing limitations in social, occupational, and personal life compounded by medical and psychological trauma [56]. Furthermore, Motamedi et al. found that women criticized the overuse of mesh surgery, inadequate consent process, and lack of a clear definition of success by surgeons, and expressed concerns about the safety of mesh products and the risk of future complications [56]. Liang et al.'s study found that sexual function deteriorated after transvaginal mesh surgery, with negative emotional reactions triggered during postoperative intercourse, particularly in the domains of pain and behavior [57]. Considering these psychosocial impacts, minimally invasive treatments such as VEL may be desirable.

Challenges in treatment selection

Currently, there are no research reports on what options should be presented to patients when complications occur after MUS surgery for SUI. Treatment selection in these cases can be complex, as it involves weighing the risks and benefits of various interventions, such as mesh removal, revision surgery, or conservative management. This bias from healthcare providers may hinder patients' decision-making, even when polypropylene mesh is inserted for the first time for conditions such as SUI. A Japanese retrospective study at multiple institutions showed that, in the treatment of stress urinary incontinence, there is a tendency to choose TVT when no other options besides mesh are provided. However, when patients are given knowledge of options other than mesh insertion, they tend not to choose mesh [46]. Bias in treatment selection by healthcare providers may hinder decision-making. Li et al.'s study found that 73.8% of patients had heard about mesh, with 55.7% citing media as their source of information. Specialized counseling significantly reduced patients' concerns (from 5.98 to 4.25, p=0.00005), and the consent rate for mesh use increased from 33.3% to 62.8% (p=0.00001) [58]. On the other hand, Tenggardjaja et al. found that while 61.8% of patients were aware of the FDA warning, 52% of them misunderstood that there had been a mesh recall, and this misunderstanding was significantly associated with information from television (odds ratio: 3.01, 95%CI: 1.28-7.06, p=0.01) [59]. Therefore, it is important to provide appropriate information and decision-making support from experts without being misled by media information. Furthermore, the effectiveness of VEL, as demonstrated by the evidence in this review, should be presented to patients as a treatment option. Studies have investigated the influence of age and menopausal status on the efficacy of VEL for treating SUI and have found significant improvements in both premenopausal and postmenopausal women [48]. VEL has also shown promising results in the treatment of SUI in young women and elite female athletes [38,39]. These findings highlight the potential of VEL treatment across various age groups, despite the potential differences in hormonal profiles and tissue regeneration processes. However, more research is needed to evaluate the long-term effects of VEL in different age groups and to understand the impact of hormonal changes on treatment outcomes, which will better guide treatment selection and patient counseling.

## Conclusions

This review provides an overview of the current evidence regarding complications following midurethral sling surgery for stress urinary incontinence and the potential of VEL treatment as a treatment option for these complications. Midurethral sling surgeries such as TVT and TOT have been widely adopted for the treatment of stress urinary incontinence. However, complications, such as persistent urinary incontinence, mesh exposure, and pain after surgery, have become problematic. Surgical treatments, such as mesh removal, have been performed for these complications; however, there are multiple complex issues, including invasiveness.

In contrast, VEL treatment has the advantages of promoting tissue regeneration, being minimally invasive, and having a low risk of complications. Research results have suggested the effectiveness of laser treatment in cases of persistent urinary incontinence after midurethral sling surgery, mesh pain, mesh erosion, and exposure. It is important to appropriately assess the indications for laser treatment and present them as a treatment option after providing sufficient information to patients. Long-term follow-up after surgery and consideration of the physical and psychological burdens are also necessary. In the future, large-scale comparative trials are required to verify the efficacy and safety of VEL treatment and to establish criteria for its indications. Laser treatment is expected to be a new treatment option for patients with complications after midurethral sling surgery.
